# Low Intrinsic Aerobic Capacity Limits Recovery Response to Hindlimb Ischemia

**DOI:** 10.3389/fcvm.2021.752955

**Published:** 2021-11-22

**Authors:** Elizabeth Granier, Madaniah O. Zakari, Musaad B. Alsahly, Lauren G. Koch, Steven Britton, Laxmansa C. Katwa, Robert M. Lust

**Affiliations:** ^1^Department of Physiology, Brody School of Medicine, East Carolina University, Greenville, NC, United States; ^2^Department of Biological Science, St. Louis Community College-Meremac, St. Louis, MO, United States; ^3^Department of Physiology, College of Medicine, Taibah University, Medina, Saudi Arabia; ^4^Department of Physiology, College of Medicine, King Saud University, Riyadh, Saudi Arabia; ^5^Department of Physiology and Pharmacology, University of Toledo, Toledo, OH, United States; ^6^Departments of Anesthesiology and Molecular and Integrative Medicine, University of Michigan, Ann Arbor, MI, United States; ^7^East Carolina Diabetes and Obesity Center, East Carolina University, Greenville, NC, United States

**Keywords:** vascular occlusion, aerobic running capacity, exercise, peripheral artery occlusion, limb ischemia

## Abstract

**Introduction:** In this study, we determined the influence of intrinsic exercise capacity on the vascular adaptive responses to hind limb ischemia. High Capacity Running, HCR; Low Capacity Running, LCR, rats were used to assess intrinsic aerobic capacity effects on adaptive responses to ischemia.

**Methods:** Muscle samples from both ischemic and non-ischemic limb in both strains were compared, histologically for the muscle-capillary relationship, and functionally using microspheres to track blood flow and muscle stimulation to test fatigability. PCR was used to identify the differences in gene expression between the phenotypes following occlusive ischemia.

**Results:** Prior to ligation, there were not significant differences between the phenotypes in the exhaustion time with high frequency pacing. Following ligation, LCR decreased significantly in the exhaustion time compare with HCRs (437 ± 47 vs. 824 ± 56, *p* < 0.001). The immediate decrease in flow was significantly more severe in LCRs than HCRs (52.5 vs. 37.8%, *p* < 0.001). VEGF, eNOS, and ANG2 (but not ANG1) gene expression were decreased in LCRs vs. HCRs before occlusion, and increased significantly in LCRs 14D after occlusion, but not in HCRs. LCR capillary density (CD) was significantly lower at all time points after occlusion (LCR 7D = 564.76 ± 40.5, LCR 14D = 507.48 ± 54.2, both *p* < 0.05 vs. HCR for respective time point). NCAF increased significantly in HCR and LCR in response to ischemia.

**Summary:** These results suggest that LCR confers increased risk for ischemic injury and is subject to delayed and less effective adaptive response to ischemic stress.

## Introduction

Peripheral artery occlusive disease (PAOD) is a major health problem with limited treatment options affecting 8-12 million Americans (1, 2). The pathophysiology of PAOD is characterized by an impaired perfusion to the lower extremities. Exercise training is one treatment option that improves quality of life and tissue perfusion characteristics, by inducing increased capillary density (3), increasing the surface area for exchange of oxygen and other substances between capillaries and muscle fibers, thereby limiting ischemic symptoms (4). Exercise training has been shown to increase capillary number within healthy skeletal muscle through the process of angiogenesis, mediated, at least in part, by vascular endothelial growth factor (VEGF) driven pathways (5–8). The mechanisms by which exercise training improves intermittent claudication in PAOD patients remains unclear (2, 9, 10). Clinical studies of PAOD indicate that patients limited by intermittent claudication who engage in any amount of weekly physical activity beyond light intensity, have a lower mortality rate than their sedentary counterparts who perform either no physical activity or only light-intensity activities (11), even after adjusting for other predictors of mortality, which include age, and body mass index (BMI). Thus, exercise training appears to induce adaptive remodeling within ischemic skeletal muscle, but the pathways and mechanisms responsible for this remodeling are still not well-characterized.

Exercise is a complex stimulus with multifactorial outcomes. These outcomes are determined by at least two genetic components (12–17). One component establishes the innate aerobic exercise capacity that each individual possesses irrespective of any subsequent training or activity (12–17), and the second genetic component establishes the pattern of responses to active exercise training (1, 2, 11, 18–20). It is estimated that up to 70% of the variation in exercise capacity are due to the genetic predisposition of an individual (12, 13). Many studies have focused on the active exercise training component (1, 2, 6, 7, 11, 18–21), but even within these studies, there is a heterogeneous response in subjects to training protocols (2, 6, 7, 11, 18–22). It is this finding that suggests the importance of investigating intrinsic exercise capacity and its impact on the response to ischemic stress.

Active exercise training in healthy individuals increases both perfusion and energy demand in the working muscle, although under strenuous exercise, the increase in demand is thought to exceed the increase in perfusion. This relative ischemic stress generates a remodeling stimulus for angiogenesis, but there must be limits because progressive loss of perfusion with advancing occlusive disease also induces a relative ischemia lower work intensity, but does not generate a comparable expansion of the vascular bed to offset it. The process of vascular remodeling is multifactorial, with vascular endothelial growth factor (VEGF) and its receptor family including VEGFR-1 [Fms-like tyrosine kinase 1 (FLT-1)], VEGFR-2 [Kinase insert domain-containing receptor (KDR) also known as fetal liver kinase (FLK-1)], and neuropilin-1 (NP-1) all participating (6, 7, 19). VEGFR-2 appears to respond following exercise training as well as with ischemic challenge independent of any active exercise (4, 19, 20). Other potential angiogenic factors that may influence intrinsic exercise capacity associated responses to ischemic challenge are angiopoietin 1 and 2 (Ang1 and Ang2) and nitric oxide (NO) (8, 18, 20, 23). Both factors are known to be involved in exercise training induced angiogenesis and ischemia induced angiogenesis, as well as the combination of both stressors. The angiopoietins are important cytokines that assist in vascular development and remodeling (24). Ang 1 promotes maturation and stabilization of vessels and is expressed widely throughout tissues, whereas Ang 2 competes with Ang 1 by displacing it from the activating Tie2 receptor. The Tie2 receptor is expressed at sites of vascular remodeling. Thus Ang1 dominance is associated with a stable vasculature while Ang2 dominance is associated with active angiogenesis (24). NO can be increased with VEGFR2 or KDR activation (24). The increase in NO production can be critical to VEGF signaling and the remodeling response of the skeletal muscle. The influence of intrinsic exercise capacity on the angiogenic response following an ischemic event has not been studied.

Low aerobic capacity or low cardiovascular fitness, is a strong predictor of early mortality within PAOD patients (2, 3, 9, 11). Koch and Britton (14–17), developed a rat model to investigate the role of intrinsic aerobic exercise capacity on responses and adaptations to chronic disease. Studies with this model show that the low endurance running capacity phenotype (LCR) rats are more prone to develop hepatic steatosis (25) and more sensitive to high fat diet-induced insulin resistance (26). These LCR rats displayed a higher incidence of both cardiovascular and metabolic syndrome risk factors than the high endurance running capacity (HCR) rats (27). Previous studies have found that the LCR phenotype has decreased capillary density when compared to the HCR counterparts (14–17, 27), which suggest that the LCR may have a decreased tolerance in hind limb ischemia models of PAOD. Studies of skeletal muscle responses to the stress of high fat feeding indicate that while the LCR's are less tolerant, the inducible responses are slower in onset but largely intact (26). However, while resting differences between the phenotypes are suggestive, it remains unknown whether the induced responses of peripheral ischemic challenge are altered by the intrinsic aerobic phenotype. Therefore, this study was designed to test the hypothesis that an increased intrinsic aerobic capacity will manifest more compensation in response to peripheral ischemia using a an established unilateral hind limb femoral artery occlusion (19, 20).

## Methods

### Animal Strains

40 LCR and 40 HCR generation 22 rats were obtained from Drs. Lauren Koch and Steven Britton at the University of Michigan. Those authors have previously described the selection process of artificial selection used to generate the HCR and LCR strains (14–17). Briefly, two-way artificial selective breeding was used to create low capacity runner (LCR) and high capacity runner (HCR) strains that were divergent for treadmill running capacity (run time to exhaustion on a graded treadmill exercise test). The 13 lowest and 13 highest running capacity rats of each sex were selected from the founder population (N: NIH stock) and randomly paired for mating. This pattern was repeated over subsequent generations to produce the divergent strains using a rotational breeding scheme for minimal inbreeding. In the present study animals from generation 22 were used. It is important to note that all the animals were housed under sedentary conditions, except for the 5 days at 11 weeks of age when the animals were phenotyped for treadmill running capacity. At all other times, animals had no exercise other than spontaneous cage activity. Once phenotyping was verified, animals were prepared for shipping at 14 weeks of age, or as soon after as weather conditions (airport tarmac temperatures <85°F) permitted. Once received by the Department of Comparative Medicine at ECU, the animals were maintained under mandatory quarantine for 10 weeks before they were released for study. Therefore, all animals were at least 24 weeks of age before they were available to be enrolled in a protocol. Rats were provided standard rat chow and water ad libitum, and were kept on a 12 h light/12 h dark time schedule until sacrifice. Animal procedures were conducted in accordance with American Physiological Society guidelines for the humane and safe use of animals, and all protocols involving animals used for these experiments were approved by the East Carolina University Animal Care and Use Committee.

### Hind Limb Femoral Artery Occlusion

Femoral artery occlusion was produced as described previously by Lloyd et al. (19, 20). Briefly, rats were anesthetized with 90:10 ketamine/xylazine solution (dosage 0.08-0.1 ml mixture per 100 g body weight *i.p*.), and the incision site was prepared with Betadine and 70% alcohol. Utilizing a small incision, the right femoral artery was isolated and three separate ligatures were placed along the femoral vascular tree. One ligature was placed 5-6 mm distal to the inguinal ligament, a second was placed on a collateral artery arising from the inguinal fat pat, and the third was placed 5-6 mm distal to the first ligature. A perfusion deficit was verified by a color and temperature in the occluded limb, compared to the contralateral limb. When all three ligatures were in place, the incision was closed in layers, analgesia was administered (Buprenex, 0.1 ml per 100 g body weight, *i.p*.) and each rat was placed on a warming blanket under a heating lamp to recover. Once spontaneous movement and sternal recumbancy were observed, the animals were placed in their cages homes and returned to the animal facility. Animals were sacrificed at 7 (18 HCR and 18 LCR) or 14 days (10 HCR and 10 LCR) following placement of the ligatures.

### Microsphere Injections

To determine relative bulk perfusion in ischemic and non-ischemic muscles, colored tracer microspheres were utilized, essentially as described in the literature in multiple studies (28, 29).

Briefly, on the day of study (7 or 14 days after occlusion) the animals were again anesthetized as described above, the right carotid artery was exposed and cannulated using a PE50 catheter, which was positioned in the left ventricle by retrograde advancement. Positioning of the catheter in the left ventricle was determined by observing the characteristic transition from arterial pressure to left ventricular pressure values and was verified postmortem by visual inspection of catheter tip placement in the left ventricle. Yellow, and persimmon (15 um) Dye-Trak microspheres (Dye-Trak; Triton Technology, San Diego, CA) were dispersed by sonication and vortex mixing. The number of microspheres for any given color (mean diameter, 15 um) injected into the left ventricle was about 900,000/0.3 ml. The microspheres were loaded into the catheter and given as a bolus injection followed by a flush of 0.5 ml of saline.

Microsphere injections were performed three times on the day of euthanasia, 7 days following surgery to occlude the right femoral artery. Yellow microspheres were injected first as a control, which allowed comparison of flow between the non-ischemic left gastrocnemius muscle, and the right, post-occlusion gastrocnemius. Following the first injection, the left femoral artery was ligated acutely, at similar locations to those used previously for the right femoral artery. Immediately after ligation, the persimmon microspheres were injected. Comparison of tracer densities was used to determine the difference between native collateral flow (left gastrocnemius) with the remodeled, post-ischemic perfusion (right gastrocnemius). Kidneys were also utilized for comparison of equal distribution and to assess approximate equal microsphere distribution between the phenotypes as the kidney weights were not different between the phenotypes.

### Microsphere Tissue Digestion and Recovery

Unless otherwise indicated, all chemicals were obtained from Sigma-Aldrich (Sigma-Aldrich Inc., St. Louis, MO). All reagent solutions were generated in-house following manufacturer's directions for microsphere recovery and processing (Dye-Trak; Triton Technology, San Diego, CA). Following the last injection of microspheres, gastrocnemius, soleus and plantaris muscles were excised and fixed in formalin for 1 h. Tissue was weighed (~1.0-2.0 g) and then subjected to tissue digestion and processing as outlined by Dye-Trak. Once a pellet of blanched microspheres and any remaining debris was obtained, the supernatant solution was used for dye analysis. Photometric absorption of each dye solution was determined by UV/Visible Spectrophotometer (wavelength 300-700 nm with 1 nm optical band width). The composite spectrum of each dye solution was resolved into the spectra of the single constituents by a matrix inversion technique, using formulas provided by the manufacturer for that purpose. The absorption spectrum of each dye was measured separately from a control sample of each colored microsphere and was used as a reference for the matrix inversion, determining the contribution of each color to the measured composite spectra at 440, 495, 545, 672 nm. In the present study, the various vascular interventions precluded access to a reference withdrawal arterial blood sample to convert microsphere count to blood flow in ml/min/g tissue. Instead, the raw counts reflect relative flow. There were no significant differences in blood pressure between any of the animals at any of the injections, there were no significant differences in renal sphere counts with any of the injections, and there were no significant differences between right and left kidneys Together, these controls suggest that there was consistent, uniform distribution of the tracers.

### High Frequency Stimulation

As a gross functional test of differences between HCR and LCR phenotypes, demand ischemia was induced by direct electrical stimulation of the gastrocnemius muscle. The pacing protocol was similar to that described by Keeton et al. (30). Briefly, high frequency electrical stimulation was accomplished using a pair of wire electrodes, ~5 mm apart, inserted directly into the body of the gastrocnemius muscle. Electrical impulses were delivered using a stimulator (Grass Instruments, Columbus OH) set to deliver repeated single pulses at a frequency of 5 Hz, 200-400 mV, and pulse duration of 1-ms duration. Preliminary experiments were used to establish the threshold voltage for stimulation response (100-200 mV), and a stimulation frequency that would produce spontaneous exhaustion of muscle contraction. The electrical stimulation protocol lasted until the muscle failed to contract with enough force sufficient to cause a visible displacement of the foot. Eight HCR and 8 LCR rats underwent high frequency pacing prior to hind limb femoral artery occlusion to establish baseline differences between the phenotypes, and 13 HCR and 14 LCR rats received pacing at the time of euthanasia to establish functional differences in the effective remodeling following femoral artery ligation.

### Rosenblatt Staining and Analysis

Gastrocnemius muscles for each animal not used for microsphere studies of blood flow were cut in half and quickly frozen. One half was placed in optimal cutting temperature (O.C.T.) compound while the other half was stored for protein isolation. The muscle in the OCT was cut into transverse sections (thickness 10 μm) and underwent capillary staining as originally defined by Rosenblatt et al. (31). Briefly, Rosenblatt staining allows capillary visualization for subsequent photographs under 20 x magnification. From the images, measurements were obtained in five fields per section, 50 myocytes/field, and in at least five separate sections, with care taken to avoid repeated sampling of the same field in sequential sections. The following indices were measured: (1) number of capillaries around a fiber (NCAF), (2) the capillary to fiber ratio on an individual fiber basis (Cap/Fi), (3) the number of fibers sharing each capillary [share factor (SF)], and (4) capillary density (CD). This stain also provides the ability to distinguish between different muscle fiber types. Slow twitch fibers were counted and are presented as a percentage of the total fiber number.

### RNA Isolation, Reverse Transcriptase-PCR, and Real-Time PCR

Total RNA was extracted from harvested gastrocnemius muscle using TriReagent (Sigma-Aldrich, USA) and cDNA was generated using the High Capacity cDNA kit from Applied Biosystems (Foster City, CA) following the manufacturer's protocol. Real-time PCR was performed using specific primers (Table 1) (Invitrogen, La Jolla, CA) and SYBR Green mix (Applied Biosystems, Foster City, CA) following the manufacturer's protocol and using GAPDH as the reference gene as CT values for this gene did not change with treatment. The Real-Time PCR detection system used was the ABI Prism 7900 sequence detection system (Applied Biosystems, Foster City, CA). LCR control samples were used for all data to be normalized with the ΔΔ^ct^ method.

**Table 1:** Real Time PCR Primers sequences.

**Primer**	**Forward sequence**	**Reverse sequence**
VEGF	TTCAAGCCGTCCTGTGTGC	TCCAGGGCTTCATCATTGC
Flt-1	CCTCGCCAGAAGTCGTATGG	CCTCGCCAGAAGTCGTATGG
KDR	TCAAGATCCTCATCCACATTGG	GGGCTTCGTGCAGGCA
Ang1	AGATACAACAGAATGCGGTTCAAA	TGAGACAAGAGGCTGGTTCCTAT
Ang2	TGGCTGGGCAACGAGTTT	TGGATCTTCAGCACGTAGCG
eNOS	GTGCTGGCATACAGAACCCA	CCATGTGGAACAGACCCCA
GAPDH	GCTGAGTATGTCGTGGAGTC	GTCAGATCCACAACGGATAC

### Statistics

Statistical analysis was performed using SPSS software (SPSS, Chicago, IL). Data are expressed as means ± SEM, and a *p*-value <0.05 was considered statistically significant. Student unpaired *t*-test were used to compare intrinsic differences between LCR and HCR run time to exhaustion and run distance. For microsphere and histology variables, simple effects (LCR control vs. LCR acute, LCR acute vs. LCR 7D, LCR 7D vs. LCR 14D, HCR control vs. HCR acute, HCR acute vs. HCR 7D, HCR 7D vs. HCR 14D, LCR control vs. HCR control, LCR acute vs. HCR acute, and LCR 7D vs. HCR 7D, LCR14D vs. HCR14D) were the comparisons of interest. A mixed model analysis of variance (2 x 3 ANOVA, two groups [LCR and HCR) x three time points (control, acute, 7D for microsphere data and control, 7D, 14D for all other data sets)] was performed using SPSS on all variables between LCR and HCR control, seven day, and 14 day endpoints. High frequency stimulation data was collected in two separate experiments, so the post ligation data was analyzed with a student *t*-test as was the pre ligation data. A generalized linear model of variances using SPSS was performed on these two data sets with the intent to assess if there was a main effect for ischemia. When there was a significant interaction, *post hoc* test were done with Bonferroni adjustment. Significant differences were accepted in all circumstances where *p*<*0.05*.

## Results

**Figure 1:**
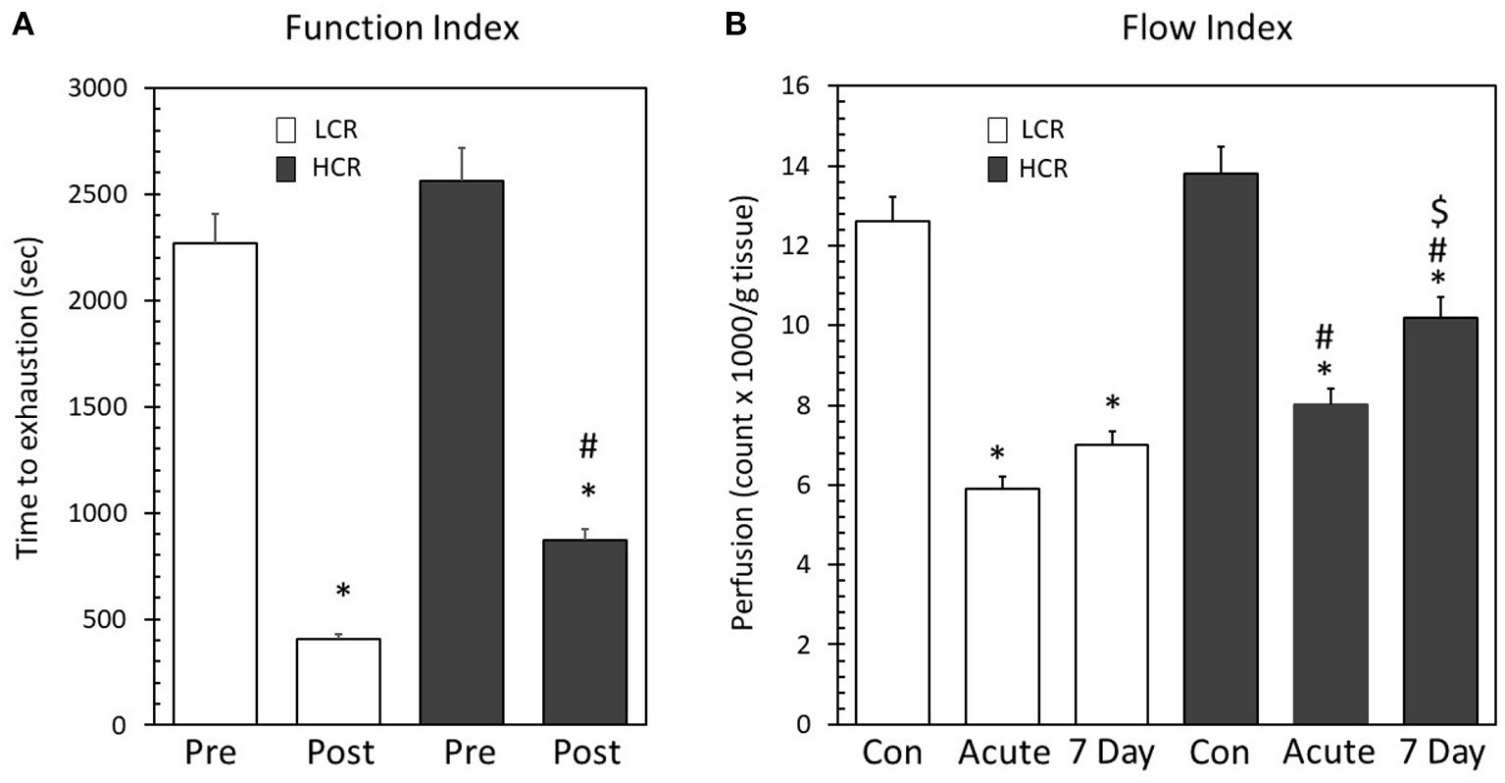
**(A)** High frequency electrical stimulation before and following placement of unilateral hind limb ligation. There was no difference between the phenotypes prior to ligation. Following ligation, the time to exhaustion decreased significantly in both phenotypes (*p < 0.05 Pre vs. Post). The LCR in post ligation time to exhaustion was significantly less than the HCR (^#^*p* < 0.05 Post LCR vs. Post HCR). **(B)** Microsphere recovery from gastrocnemius, soleus, and plantaris muscles. Both phenotypes displayed significantly decreased blood flow following ligation (*p < 0.05 vs. phenotype control). LCR microspheres/g tissue was significantly less than the HCR at both the acute and 7D ischemic time points (^#^*p* < 0.05 vs. same time point between phenotypes). Following 7 days, the LCR did not significantly alter blood flow while the HCR significantly increased blood flow from acute (^$^*p* < 0.05 vs. 7 day time point within phenotype).

To provide a functional measurement for comparison between the phenotypes, high frequency electrical stimulation of the gastrocnemius muscle was performed prior and following ligation in two separate experiments. Prior to ligation, there were not significant differences between the phenotypes in the time to exhaustion with high frequency pacing [LCR time (s) = 2,291 ± 186 vs. HCR time (s) = 2,571 ± 218]. Following ligation, both strains decreased the time to muscle exhaustion with pacing, consistent with having induced an injury, but there was a significant difference between the strains (LCR = 437 ± 47 vs. HCR = 824 ± 56). The LCR gastrocnemius muscle time to exhaustion was significantly less than the HCR (Figure 1A), suggesting greater functional impairment in the LCR as a result of the occlusion.

Similar to the findings with pacing, there also were not differences in resting perfusion (LCR number of microspheres/g tissue = 13,424 ± 688 vs. HCR number of microspheres/g tissue = 12,563 ± 823). Not surprisingly, ligation of the femoral artery significantly decreased perfusion in both the LCR (6,383 ± 366) and HCR (7,821 ± 424) strains, but the decrease in perfusion was more severe in the LCR phenotype both immediately after ligation and 7 days after ligation (LCR = 6,880 ± 513 vs. HCR 9,522 ± 721) (Figure 1B). These data indicate that while both phenotypes decreased tissue perfusion in response to the hind limb ligature, the extent of perfusion deficit, and the resulting pattern of recovery over the next seven days was significantly different as a function of the phenotype.

**Figure 2:**
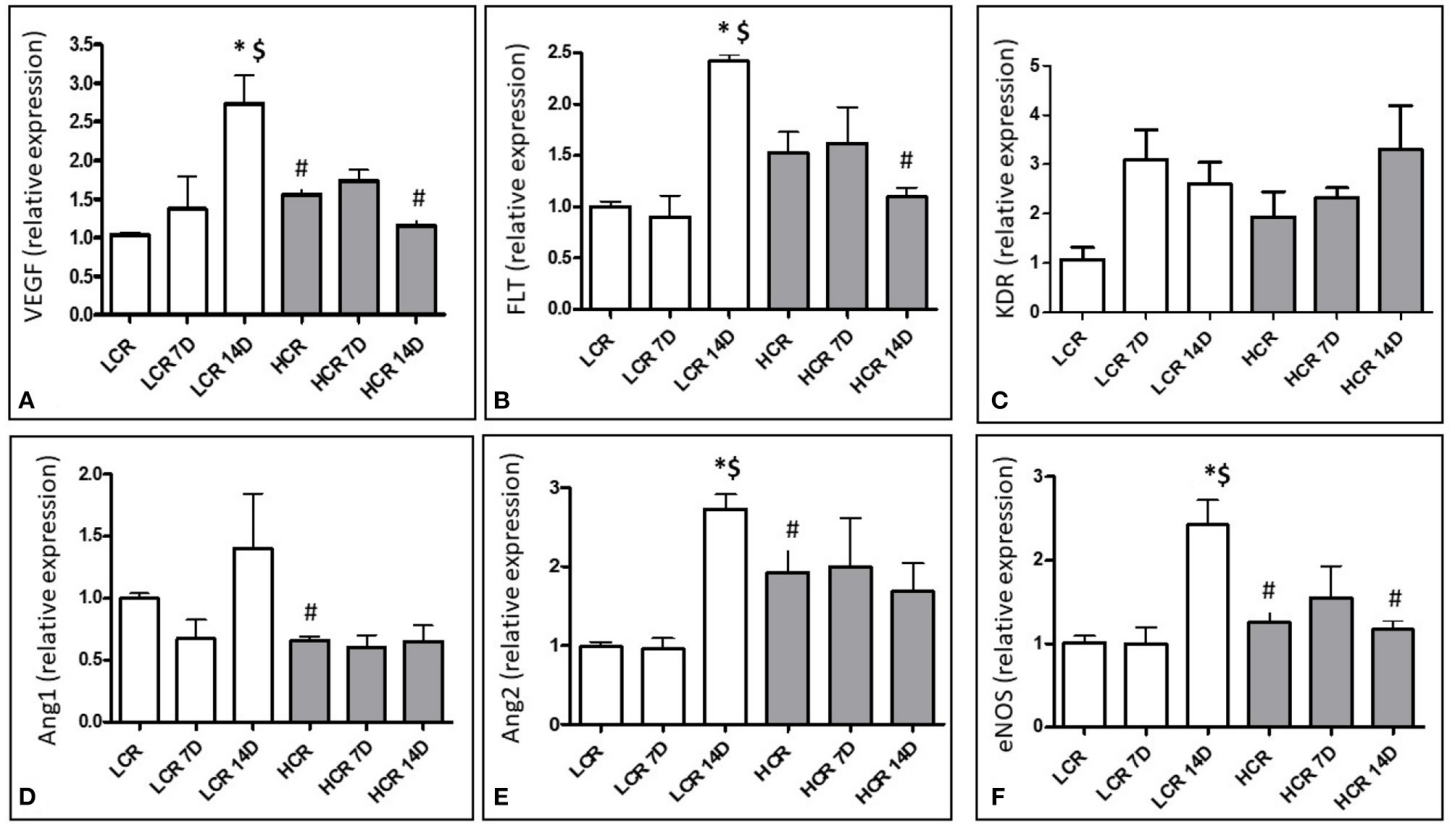
Relative mRNA expression for VEGF **(A)**, Flt-1 **(B)**, KDR **(C)**, Angiopoietin 1 **(D)**, Angiopoietin 2 **(E)**, and eNOS **(F)**. *p < 0.05 vs. control within phenotype; ^#^*p* < 0.05 vs. same time point between phenotypes; ^$^*p* < 0.05 vs. 7 day time point within phenotype.

mRNA expression for angiogenic growth factors was assessed with quantitative real time PCR (qRTPCR). VEGF expression was significantly lower in the LCR phenotype under baseline (pre-ischemic conditions (Figure 2A). Neither the LCR nor the HCR animals demonstrated significantly altered VEGF mRNA expression following 7D of ischemic challenge and baseline differences disappeared between the phenotypes at this time point. 14 days after ligation, the LCR significantly increased VEGF mRNA expression in the gastrocnemius muscle compared to both control (^*^Figure 2A) and 7D ischemic levels (^$^Figure 2A). Moreover, VEGF mRNA expression also was significantly greater in the LCRs than the HCRs 14 days after occlusion (^#^Figure 2A).

In contrast to VEGF, neither VEGF receptor (Flt-1) mRNA nor VEGF receptor (KDR) mRNA expression was significantly different between the phenotypes under baseline, pre-ischemic conditions. Neither the LCR nor the HCR animals demonstrated significantly altered Flt-1 or KDR mRNA expression following 7D of ischemic challenge. Similar to VEGF, Flt-1 mRNA expression increased significantly in the LCR ischemic muscle at 14 days, compared to either control (^*^Figure 2B) or 7D ischemic levels (^$^Figure 2B) and was also significantly greater than expression in the HCRs at 14 days post ischemia (^#^Figure 2B). However, these changes exhibited in Flt-1 were not in KDR mRNA expression (Figure 2C).

Angiopoietin 1 mRNA expression was significantly higher in the LCR phenotypes under baseline conditions (^#^Figure 2D) but neither phenotype significantly altered the expression of this growth factor and the differences present at baseline were no longer present at 7D or 14D.

Angiopoietin 2 mRNA expression was significantly higher in the HCR phenotype under baseline conditions (^#^Figure 2E), and did not change in this phenotype at either 7 or 14 days after occlusion. In contrast, the LCR phenotype showed significant increases in Ang 2 expression 14 days after occlusion (^*,$^Figure 2E), abolishing any differences that were present between the phenotypes before occlusion.

**Figure 3:**
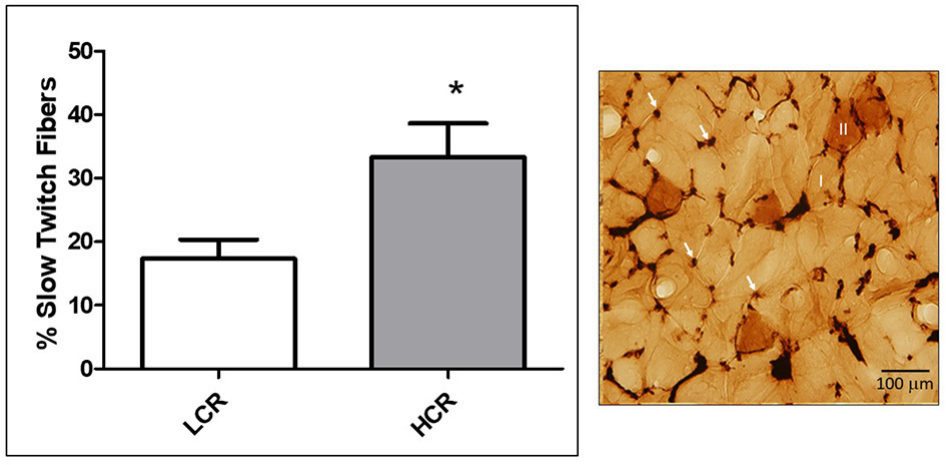
Sample image of Rosenblatt attained muscle cross section (right). Arrows indicate capillaries and numerals indicate either type II (dark stained) or type I (light stained) muscle fibers. Left is a graphical summary showing the fraction of slow twitch muscle fibers within gastrocnemius muscle under baseline conditions. LCR had a significantly smaller percentage of slow twitch fibers compared to HCR (*p < 0.05).

eNOS mRNA expression was significantly lower in the LCR phenotype under baseline conditions (^#^Figure 2F). Neither the LCR nor the HCR animals demonstrated significantly altered eNOS mRNA expression following 7D of ischemic challenge and baseline differences disappeared between the phenotypes at this time point. 14 days after ligation, the LCR significantly increased eNOS mRNA expression in the gastrocnemius muscle compared to both control (^*^Figure 2F) and 7D ischemic levels (^$^Figure 3). Moreover, eNOS mRNA expression also was significantly greater in the LCRs than the HCRs 14 days after occlusion (^#^Figure 2F).

Histology data indicate that the LCR has reduced capillary density (CD) (LCR = 417.81 ± 23.6) compared to the HCR in non-ischemic gastrocnemius muscle (HCR = 614.6 ± 57.5) (^#^*p* < 0.05, Table 2), consistent with previous reports (3, 28). The HCR phenotype increased capillary density at both 7 (918.54 ± 197.6) and 14 days (1,015.41 ± 174.9) after occlusion (^*^Table 2). In contrast, capillary density in LCRs was significantly lower at all time points (LCR 7 Day = 564.76 ± 40.5, LCR 14 Day = 507.48 ± 54.2) (^#^Table 2), and in contrast to HCRs, capillary density did not change in LCRs after occlusion. There were no differences in capillary contacts per fiber (NCAF) between phenotypes under control conditions (LCR = 4.547 ± 0.220 vs. HCR = 4.280 ± 0.217), but both phenotypes increased NCAF significantly in response to ischemia. However, the increase in the HCRs was earlier in onset, present at both 7 (5.170 ± 0.276) and 14 days after occlusion (5.210 ± 0.255) (^*^Table 2), while the LCR was significantly elevated only at the 14 day endpoint (6.220 ± 0.481) (^*^Table 2). At 14 days, the NCAF values had become significantly greater in the LCR compared to the HCR animals (^#^Table 2).

**Table 2:** Skeletal muscle morphology and capillarization in the gastrocnemius muscle of control, 7 and 14 day ischemic tissue of LCR and HCR rats.

	**LCR**	**HCR**
Area μ*m*^2^		
Non-ischemic gastrocnemius	4349.8 ± 281.3	2783.6 ± 236.3^#^
7 day ischemic gastrocnemius	3031.4 ± 372.7^*^	2433.3 ± 369.8
14 day ischemic gastrocnemius	4938.5 ± 788.6^$^	2292.7 ± 622.2^#^
Perimeter μm		
Non-ischemic gastrocnemius	267.76 ± 8.57	211.85 ± 8.75
7 day ischemic gastrocnemius	224.57 ± 9.44	200.72 ± 14.4
14 day ischemic gastrocnemius	290.61 ± 26.95	190.29 ± 22.9
Capillary density, capillaries/mm^2^ (CD)		
Non-ischemic gastrocnemius	417.81 ± 23.6	614.60 ± 57.5^#^
7 day ischemic gastrocnemius	564.76 ± 40.5	918.54 ± 197.6^*^^#^
14 day ischemic gastrocnemius	507.48 ± 54.2	1015.41 ± 174.9^*^^#^
Capillary contacts (NCAF)		
Non-ischemic gastrocnemius	4.547 ± 0.220	4.280 ± 0.217
7 day ischemic gastrocnemius	4.370 ± 0.584	5.170 ± 0.276^*^
14 day ischemic gastrocnemius	6.220 ± 0.481^*^^$^	5.210 ± 0.255^*^^#^
Individual capillary to fiber ratio (cap/fib)		
Non-ischemic gastrocnemius	1.662 ± 0.089	1.555 ± 0.073
7 day ischemic gastrocnemius	1.603 ± 0.233	1.896 ± 0.149^*^
14 day ischemic gastrocnemius	2.248 ± 0.183^*^^$^	1.852 ± 0.097^*^^#^
Share factor (SF)		
Non-ischemic gastrocnemius	2.896 ± 0.014	2.933 ± 0.062
7 day ischemic gastrocnemius	2.890 ± 0.038	2.938 ± 0.069
14 day ischemic gastrocnemius	2.935 ± 0.023	3.14 0.015

# *Significant differences between phenotypes at corresponding time point*.

* *Significant differences from control condition within phenotype*.

$ *Significant differences vs. 7 day ischemic value within phenotype*.

Similar to the NCAF results, the capillary to fiber ratio on the individual fiber basis (Cap/Fib) were not different under baseline conditions (LCR = 1.662 ± 0.089 vs. HCR = 1.555 ± 0.073), but increased in both phenotypes in response to occlusion. Again, the increase in the HCR phenotype was evident at both the 7 (1.896 ± 0.149) and 14 day (1.852 ± 0.097) time points compared to control (^*^Table 2), but a similar increase above control levels was not seen in the LCR animal until 14 days (2.248 ± 0.183) (^*^Table 2), at which point the NCAF values were significantly higher in the LCR than the HCR animals (^#^Table 2).

The percentage of slow twitch muscle fibers in the mixed gastrocnemius muscles from the LCR and HCR animals was compared (Figure 3). The LCR rats had a significantly lower percentage of slow twitch fibers in the control gastrocnemius muscle compared to the HCRs (^*^p < 0.05, Figure 3).

## Discussion

The development of intermittent claudication is a major decrement to the quality of life in patients (1, 2, 31–33). A mounting body of evidence suggests that exercise training can increase perfusion in ischemic tissue, improving quality of life and avoiding the necessity for surgical revascularization and other expensive therapies (1–3, 9, 33, 34). The majority of research on aerobic exercise training in PAOD patients has examined its effects upon vascular remodeling, including the mechanisms underlying changes in capillary density within the ischemic muscle (6, 7, 21, 22). However, many patients with intermittent claudication are unable to exercise or participate in exercise protocols due to pain in the ischemic limb or arthritic problems in joints, and exercise studies do not always provide definitive remodeling within tissue. Developing a better understanding of the genetic influences associated with intrinsic aerobic capacity and endurance capacity on the vascular remodeling process in response to ischemic challenge may help explain the variability in PAOD patients responses to aerobic exercise, and may provide new treatment options for individuals unable to participate in aerobic exercising protocols. The goal of this study was to investigate whether rats with low intrinsic running capacity have different vasculogenic responses following peripheral arterial occlusion than rats with high intrinsic running capacity.

The results of this study demonstrate that rats selectively bred, but not trained, for high endurance running capacity (HCR) differ in their response to ischemic stress in a model of PAOD compared to the low endurance running capacity (LCR) counterparts. These results provide strong evidence indicating that low endurance running capacity confers increased risk for ischemic injury and is subject to delayed and less effective adaptive response to ischemic stress.

LCR and HCR's were inherently different in capillary density before ischemia, with HCR rats having significantly higher capillary density than LCR rats. Under baseline conditions, the HCR strain also showed a higher relative expression of mRNA for VEGF, eNOS, Ang2 and significantly less relative expression of Ang1 compared to the LCR strain. These results are consistent with those found by Lloyd et al. (19), who showed similarly increased amounts of angiogenic markers in response to active aerobic exercise training protocols in rats, except that in the present studies, these were intrinsic differences predicated only on intrinsic aerobic phenotype, and not dependent on any active training component. Similar to the actively exercised rats, the HCRs appear to have an underlying increased angiogenic potential under basal conditions compared to the LCR phenotype.

LCR muscle cells showed a significantly larger area compared to the HCR, a finding consistent with Howlett et al. (35) who found that mean cross-sectional area of the HCR fiber was 35% lower compared to the LCR. The combination of smaller fiber area, more capillaries, and greater oxidative capacity in HCR rats may provide the basis for explaining the inherent higher VO_2max_ observed in these animals without aerobic exercise training, and would be consistent with increased resistance to ischemic injury.

Increased capillary density (CD) (capillaries/mm^2^) has been shown to strongly correlate with increased skeletal muscle oxygen conductance in this model (25). We now extend those observations by demonstrating that the HCRs may have inherently better protection for maintenance of tissue perfusion under ischemic conditions. This was functionally evident with the prolonged time to exhaustion with high frequency electrical stimulation, and by higher post-ischemic perfusion levels. Although the initial drop in blood flow after occlusion is greater in the LCRs, consistent with provoking a more potent ischemic stress, the LCRs do not demonstrate a measurable recovery in perfusion at seven days. The HCRs initially lose less perfusion with acute arterial occlusion, presumably associated with better ischemic tolerance, and also recover perfusion in the post-ischemic tissue, consistent with anatomic remodeling, altered arteriolar/collateral tone favoring dilation, or both. This flow preservation, suggesting an improved tolerance for ischemic stress by the HCR, is further supported by the HCR's ability to maintain muscle fiber area (Table 2). Furthermore, despite less ischemic pressure and negligible changes in fiber area, HCR rats showed a significant increase in capillary density at seven days despite negligible changes in the mRNA expression of angiogenic markers suggesting the possibility that the elevated baseline expression levels of the typical angiogenic growth factors in the HCRs were sufficient to drive any required angiogenesis. These might be more analogous to patients who develop claudication, but do not progress to critical limb ischemia.

Conversely, the LCR animals did not generate a measurable change in perfusion following seven days of ischemic pressure. These animals responded to seven days of femoral artery ligation with a significant loss of muscle fiber area consistent with atrophic loss of muscle mass. Perhaps because there was loss of muscle mass, there was a reduced ischemic signal source such that the LCR rats did not alter capillary density (CD), Cap/Fib ratio, or NCAF significantly from baseline levels, and perfusion did not recover. The LCR rats did not show significant changes in mRNA expression for any of the angiogenic factors from the baseline to the 7D ischemic time point, suggesting that the LCR rats initially respond to seven days of ischemia was a loss in fiber area and no angiogenic response, despite a substantial loss in perfusion. These might be more analogous to patients who develop critical limb ischemia.

Following 14 days of femoral artery occlusion, the HCRs continued to maintain fiber area but did not significantly alter any angiogenic markers from seven day levels, suggesting that after 14 days of ischemia, angiogenesis was no longer an active pathway within this phenotype and vascular remodeling processes had been completed. This finding is consistent with Lloyd et al. who reported that ischemia-induced angiogenesis typically alters mRNA within 7-10 days and returns toward baseline levels by about 14 days (19). Of particular interest in the present study was that HCRs appeared to increase the percentage of slow twitch fibers with 14 days of ischemia. A fast to slow fiber switch is normally seen in response to aerobic exercise training and aging (19), but ischemia and unloading is typically associated with a slow to fast phenotypic shift. The dynamic recovery response over a 2 week period in the HCRs appears to involve a shift in fiber type and an increase in fiber numbers, associated with higher pre-existing angiogenic signals and fueled by a better flow recovery. In contrast, after an initial fiber loss at 7 days, muscle fiber area in the LCRs significantly increased again 14 days after femoral artery occlusion, suggesting that there was a change in fiber size, but not fiber number, and an entirely different remodeling response. The increase in muscle fiber size may have been sufficient to recover a relative ischemia signal, as significant increases in mRNA expression for VEGF, Flt-1, eNOS, and Ang2 were observed at 14 days after occlusion. Together, these findings suggest that an angiogenic drive remains inducible in the LCR phenotype, but that the dynamic between muscle mass, muscle number, demand ischemia and vascular remodeling still is incompletely understood. Recent reports in mice (36), and subsequently in humans (37), have suggested a potential role for BAG-3 (Bcl-2-Associated Athanagene-3) coding variants in dictating the response to ischemia in skeletal muscle. This angiogenic stimulus may be in response to signals associated with hypertrophy of the LCR cells, but not with the occlusive stimulus, or may be occurring at 14 days as a synergistic action of the loading stimulus in combination with ischemic drive within the tissue.

The present study suggests that LCR rats have an altered response to hind limb femoral artery occlusion compared to the HCR rats. This altered response is partially due to the phenotypic differences under baseline conditions, including significantly less capillary density, less angiogenic factor potential, larger fiber area, and a smaller percentage of slow twitch muscle fibers within the gastrocnemius muscle in the LCR animals. With onset of occlusion, the LCRs also appear to have an altered inducible response compared to the HCR. These alterations include fiber area initially decreasing, followed by a rebound to a larger area, a potent angiogenic upregulation at 14D ischemia and no significant changes in the percentage of slow twitch muscle fibers. These data suggest that low intrinsic aerobic capacity may have increased injury associated with ischemic disease and will generate an altered response that may require a combination ischemic challenge, and signaling from some other stress, such as increased mechanical loading of the muscle, to adequately stimulate vasculogenic responses.

## Data Availability Statement

The raw data supporting the conclusions of this article will be made available by the authors, without undue reservation.

## Ethics Statement

The animal study was reviewed and approved by Institutional Animal Care and Use Committee (IACUC) East Carolina University.

## Author Contributions

EG and RL participated in designing, conducting, data analysis, and writing. MA and MZ participated in data analysis and writing. SB and LGK provided the animals and participated in experimental design. LCK participated in data analysis and manuscript review. All authors contributed to the article and approved the submitted version.

## Funding

This study was supported by NIH grant number P40OD021331, National Institutes of Health, Office of Research Infrastructure Programs.

## Conflict of Interest

The authors declare that the research was conducted in the absence of any commercial or financial relationships that could be construed as a potential conflict of interest.

## Publisher's Note

All claims expressed in this article are solely those of the authors and do not necessarily represent those of their affiliated organizations, or those of the publisher, the editors and the reviewers. Any product that may be evaluated in this article, or claim that may be made by its manufacturer, is not guaranteed or endorsed by the publisher.
